# The Relationship Between Quality of Work Life and Job Satisfaction of Faculty Members in Zahedan University of Medical Sciences

**DOI:** 10.5539/gjhs.v7n2p228

**Published:** 2014-10-28

**Authors:** Fatihe Kermansaravi, Ali Navidian, Shahindokht Navabi Rigi, Fariba Yaghoubinia

**Affiliations:** 1 Pregnancy Health Research Center, Zahedan University of Medical Sciences, Zahedan, Iran

**Keywords:** quality of work life, job satisfaction, faculty members

## Abstract

**Background:**

Quality of work life is one of the most important factors for human motivating and improving of job satisfaction.

**Aim:**

The current study was carried out aimed to determine the relationship between quality of work life and job satisfaction in faculty members of Zahedan University of Medical Sciences.

**Method:**

In this descriptive-analytic study, 202 faculty members of Zahedan University of Medical Sciences in 2012 were entered the study through census. The job satisfaction questionnaire of Smith and Kendall and Walton Quality of Work Life questionnaire were used for data collection. Validity and reliability of questionnaires were confirmed in previous studies. Data analysis was done using SPSS 18. The Pearson correlation coefficient and multiple regression tests were used for data analysis.

**Result:**

The mean score of quality of work life was 121/30±37/08 and job satisfaction was 135/98±33/78. There was a significant and positive correlation between job satisfaction of faculty members and their quality of work life (P=0.003). In addition, two components of quality of work life “adequate and fair compensation” (β=0.3) and “Social Integration” (β=0.4) can predict job satisfaction of faculty members.

**Conclusion:**

According to correlation between job satisfaction and quality of work life in faculty members, job satisfaction can be improved through the changing and manipulating the components of quality of work life and in this way; the suitable environment for organization development should be provided.

## 1. Background

Quality of work life is a comprehensive concept which is consisted of physical and psychological health, economic situations, personal belief and interaction with environment (Khorsandi et al., 2010). In contemporary management, the concept of quality of work life has been changed to a social issue, while in the past decades only the personal life was emphasized (Mirkamali & Narenji Sani, 2008). The meaning of quality of work life is subjective imagination and the perception of organization personnel about the physical and psychological desirability of work environment and their work situations (Yavari, Amir Tash, & Tondnevis, 2009).

Walton (1973) defined the quality of work life as the personnel reaction to work; especially its essential outcome in relation to job needs satisfaction and psychological health. According to this definition, quality of work life emphasizes on personal outcomes, work experiences and how to improve the work in order to meeting the personal needs. Walton presented a theoretical model for explaining the quality of work life that is consisted of adequate and fair compensation, safe and healthy environment, constitutionalism in organization, preparing the opportunity for continued growth and security, social relevance of work life, social integration, development of human capabilities and the total life space (Walton, 1973).

From 1980 to 2006, many researches have been done about quality of work life and results showed that there is positive relationship between quality of work life and some variables in organization such as job satisfaction. Job satisfaction has been defined as idea, perception and positive attitudes and emotions of individuals about profession which are affected by some factors such as work environment, organizational system, work environment relationship and socio-cultural factors (Mirkamali & Narenji Sani, 2008; Armstrong, 2006).

Qualitative and quantitative researches show that the most experts have consensus on factors such as work situations and work type, interactions with manager and colleagues, ways of preferment in organization, salary and benefit as the major predictor factors of job satisfaction (HongLu, While, & Barriball, 2007).

The mission of quality of work life is the creating of job satisfaction for personnel and helping to organization for employee selection and retention. Quality of work life is one of the most important factors for human motivating and improving job satisfaction (Royuela, Jordi, & Jourdi, 2009).

University as the most important educational and research center has an effective role in community promotion toward educational, social, cultural and economic goals and the faculty member is one of the main components of educational system. Supporting of human resources and helping to appropriate efficiency of these resources and academic elite is the guarantee of community development and progress (Oplatka, 2009).

According to expanded duties of faculty members such as training of skilled and committed human resources, research, therapeutic services, executive activity and personal development, attention to this topic is very important (Eklund, 2008).

Previous studies showed that there is positive relationship between job satisfaction and quality of work life. In study which was conducted about faculty members in Bangladesh, results showed that all of the aspects of quality of work life have a positive relationship with job satisfaction (Tabassum, 2012).

Results of Ballou’s study (2007) also showed that the satisfied employees work with favorites in work environment and are loyal to organization and help to increase the efficiency and capital in organization (Ballou, Norman, & Goodwin, 2007). Results of other studies in Columbia also showed that the faculty’s knowledge of work life has strong and direct impact on satisfaction or incentive to withdraw from the university (Dolan et al., 2008).

This relationship also has been reported in studies which were done in Iran (Mirkamali & Narenji Sani, 2008; Teymouri, 2008). In study by Soltanzadeh (2012), components such as constitutionalism in organization, social relevance of work life, social integration were the best predictors of job satisfaction in faculty members (Soltanzadeh, Ghalvandy, & Fatahy, 2012).

In universities in Iran, formal employment and job security changed to stressful issues because of some factors such as the long process of faculty recruitment, scholarships allocation, stated the need. Workload and teaching hours of faculty also hinder some activities in other areas such as research and service (Noor Shahi & Samie, 2011). Many studies have been done in other countries about job satisfaction of faculty members.

But, the conducted studies about quality of work life in Iran are less and limit, thus it is necessary that the more and comprehensive research conduct in this topic. It is very important especially when the increase of quality of work life and its relationship with job satisfaction is propounded.

No study has been done concerning of quality of work life and job satisfaction in faculty members in Zahedan University of medical sciences. Moreover; according to importance of these two variables for advancing the academic goals and creating more efficiency and also some situations in Sistan & Baluchestan province in Iran such as shortage of amenities, this study conducted aimed to determine the relationship between quality of work life and job satisfaction in faculty members.

## 2. Method

In this descriptive-analytical study, all of the faculty members who were 202 people and were employed in Zahedan University of medical sciences in 2012 entered to study through census. The contract, committed to serving and tuition teachers were excluded from the study. Data were gathered through information form (age, gender, marital status, work experience, academic degree and employment status) and the Walton quality of work life questionnaire (Walton, 1973) and Job Descriptive Index by Kendall and Smith (Smith, Kendall, & Hulin, 1969).

Quality of work life questionnaire is consisting of 40 items which has been designed in 5-point Likert scale (never 1, rarely 2, sometimes 3, most of time 4, always 5). 5 items are related to adequate and fair compensation, 5 items are about safe and healthy work environment, 5 items are about constitutionalism in organization, 5 about preparing the opportunity for continued growth and security, 5 items are related to social relevance of working life, 5 about social integration, 5 about development of human capabilities and 5 about the total life space.

The score of each item is from 1 to 5. Thus, the overall score of this questionnaire will be in range of 40–200. Validity and reliability of questionnaire was confirmed in many studies and α=0.92 has been reported (Mirkamali & Narenji Sani, 2008; Yavari, Amir Tash, & Tondnevis, 2009). Job Descriptive Index questionnaire was made by Kendall in 1969 and it is one of the most common tools for measuring job satisfaction. This scale assess the 5 various aspects of job satisfaction.

There are some criteria for each aspect that measure the individual’s feelings about job. The five aspects of this index are consisting of five scales of work type (10 items), supervisor or manager (10 items), colleagues (10 items), preferment in organization (5 items) and salary and benefit (6 items).

Score of each item is in range of 1–5. Thus; the overall scores of job satisfaction varies in range of 41-205. The more score of this questionnaire is indicative of the more job satisfaction. The internal consistency of this questionnaire was reported as 0.89–0.92 by Kendal and Halin (Smith, Kendall, & Hulin, 1969). This questionnaire was used in many studies in Iran and its validity and reliability was confirmed (Heydari, 1998; Ranjbar, & Vahidshahi, 2007).

In study by Zamini and Hoseyninasab, this questionnaire was completed by 20 people and chronbach’s alpha=0.92 was obtained for the whole of questionnaire and for subscales was 0.85, 0.96, 0.92, 0.87 and 0.84 respectively (Zamini & Hoseyninasab, 2008). The mean score was calculated for each of the components of jab satisfaction and also total score of questionnaire. In current study questionnaires were delivered to faculty members by educational employees for completing during three weeks and then were returned to researcher.

Ethical considerations were adhered, informed consent was obtained and assurance of confidentiality and anonymity was done. Data analysis was done using SPSS 18. Central tendency indexes were used for description of quantitative data and for qualitative variables, frequency and percentage were used. Also, Pearson correlation coefficient and multiple regression were used for measuring the correlation between quality of work life and job satisfaction.

## 3. Results

The mean age of faculty members was 40.91±8 with range of 28–59 years old and the mean of their work experience was 11.9±8.14 with range of 1–33. Demographic characteristics of subjects are presented in Table 1.

**Table 1:** Demographic characteristics of faculty members

Variable	Number	Percent
**Marital status**		
Single	32	15.9
Married	168	84.1
**Gender**		
male	113	55.9
Female	89	44.1
**academic degree**		
educator	70	34.6
Assistant professor	103	51
Associate professor	25	12.4
professor	4	2
**employment status**		
Formal employment	27	13.4
Permanent employment	74	36.6
contract employment	83	41.1
**inhabitancy status**		
native	132	67.7
Non-native	61	31.3

The mean score of quality of work life was 121.3 (SD=37.08). The highest mean was related to development of human capabilities (18.78±4.5) and the least mean was for constitutionalism in organization (12.84±3.92). The mean score of job satisfaction was 135.98±33.78 and the highest mean was related to job characteristics aspect (36.65±7.29) and the least mean was for ways of preferment (16.44±5.19)

A direct and significant correlation was found between quality of work life and job satisfaction of faculty members (r=0.23, p=0.003). Also, there was direct and significant correlation between aspects of social relevance, the total life space and social integration with job satisfaction (Table 2).

**Table 2:** Correlation between quality of work life score and its components with job satisfaction

Job satisfaction	Variable	

	r	p
**Quality of work life**	0.23	0.003
**Social relevance**	0.17	0.01
**The total of life space**	0.22	0.002
**Social integration**	0.25	<0.001

According to significant values (p=0.000, F=4.92) and the multiple regression analysis about components of quality of work life with job satisfaction, it can be concluded that the regression model was composed of 8 independent variables (aspects of quality of work life) and 1 dependent variable that can explain the changes in job satisfaction.

Based on Multiple regression equations (Y=a+b_1_x_1_+b_2_x_2_+……b_k_x_k),_ R^2^ value shows that the 17 percent of all of the changes in job satisfaction are associated to 8 independence variables in this equation (aspects of quality of work life). In other words, set of independent variables predict the 17 percent of variance of job satisfaction.

β Coefficient (regression coefficients) is indicated that the adequate and fair compensation (β=0.3) and social integration (β=0.4) are predictors of job satisfaction (Table 3).

**Table 3:** Multiple regression analysis of each component of quality of work life with job satisfaction 1^1^, 2^2^

Predictor variables	β	p
Fair compensation	0.301	0.001
safe and healthy work environment	0.07	0.35
Opportunity for Continued Growth	0.12	0.13
Constitutionalism in the work organization	0.26	0.17
Social relevance	0.10	0.24
The total life space	0.17	0.07
Social integration	0.40	<0.001
Development of Human Capacities	-0.10	0.21

1 Criterion variable: Job satisfaction.

2 R^2^= 0.17

## 4. Discussion

According to study results, there is positive and significant relationship between quality of work life and job satisfaction which was indicating that better quality of work life is associated with more job satisfaction in faculty members.

Results of studies which have been done in Iran such as Soltanzadeh et al. (2012), Zakerian et al. (2012), Shahbazi et al. (2011), Heidarie et al. (2010), Goudarznand-Chegini et al. (2010), Saedi et al. (2010), Mirkamali and NarenjiSani (2008) corroborated the results of current study.

Also, the results of studies in other countries such as HongLu (2007), Hua (2006), Conklin (2008), Heinonen and Sarima (2009), Adhikari (2010) and Rose (2006) confirmed the relationship between quality of work life and job satisfaction (HongLu, While, & Barriball, 2007; Hua, 2006; Conklin, 2008; Heinonen, & Saarimaa, 2009; Adhikari, & Gautam, 2010; Rose et al., 2006).

Recognition of related factors with job satisfaction in faculty members is very important, because we can increase job satisfaction and prepare the conditions in order to organizational growth with manipulating and changing the components of quality of work life. The existence of components of quality of work life provide situation for satisfaction and peace, responsibility, optimal use of Physical space and educational tools for faculty members.

The study by Conklin (2008) showed that there is a positive and significant relationship between quality of work life and job satisfaction and its changes (Heinonen & Saarimaa, 2009). Results of study in Europe (2008) concerning the health and management efficiency demonstrated that the style of work life as a psychological factor in work environment can increase the efficiency of staff (Kirsten, 2008).

The results of current study about relationship between components of quality of work life and job satisfaction showed that there is a direct and significant relationship between aspects of social dependency, life space and social integration. Zakerian (2012), Hosseini (2008), Othman (2009) and Lawler (2007) stated a significant relationship between aspects of work life and job satisfaction (Zakerian et al., 2014; Hosseini et al., 2008; Othman, & Mok, 2009; Lawler, Chan Huang, & Yilei, 2007).

According to direct relationship of component of social relevance which is referred to the perception of faculty about social responsibility in system and managers valuing of community rules, thus; it can be created a sense of duty through participation of members in decision making and team working. Also, it can be realized this aspect with using of faculty’s thought and idea in policy.

Thus, the organizations can improve all of the aspects of quality of work life in order to increase of job satisfaction. They can create the situations in work environment for demonstrating the abilities and creativities of staff and create proper opportunities for success, safety, job preferment and staff dynamics.

The aspect of work life space can be strengthened through establishment a balance between work life and other parts of life such as leisure time, education, family life and; with reduction of sensible and insensible job stress.

Results of regression analysis which was done in order to prediction of job satisfaction through the components of quality of work life showed that social integration and adequate and fair compensation are the most important predictors of job satisfaction respectively.

In other studies; constitutionalism, social integration and preparing the opportunity for continued growth and security (Mirkamali & NarenjiSani, 2008), professional satisfaction and status of life space (Zakerian et al., 2014) and organizational climate (safety work environment, providing job opportunities) were as the most important predictors of job satisfaction. Lawler et al. (2007) believed that the safe and healthy work environment, adequate and fair compensation; and job security have positive effect on organizational commitment (Lawler, Chan Huang & Yilei, 2007). The reasons of some differences between results of previous studies with current study are the difference in research population, level of education or difference in data collection methods.

Social integration was one of the factors affecting on quality of work life in faculty members. According to study results, the faculty members were dissatisfied about the work space that is the most important factor in encouraging them for doing their duties and responsibilities and creating a sense of belonging to the organization.

As a result, it is recommended that the university encourage faculty members to creativity and innovation through the preparing of appropriate context for teaching and supporting them by colleagues and managers.

Judging faculty members about objective and observable conditions of organization is affected by their perception and interpretation of organizational environment. Thus; the university can strengthen the sense of belongingness to organization in faculty members through the creating of appropriate and positive working climate and also, can create the proper perception about social responsibility in organization through the using of their expertise and experiences for problem solving in organization.

The next influential factor in job satisfaction with the least mean in both groups of basic science and clinical faculty was fair compensation. Result showed that there is no a proper balance between workload and consumed time and energy and also community standards in financial payment system from viewpoint of faculty members. Thus; it is necessary to revise the appropriateness of salary according to community situations concerning standards and inflation.

## 5. Conclusion

According to study results, quality of work life has the predictability of job satisfaction of faculty members and can be effective in improving their job satisfaction. Thus; job satisfaction can be improved through the changing and manipulating of the quality of work life components. The universities can increase the job satisfaction in faculty members through the proper program such as monetary and non-monetary rewards, creating opportunity for optimal use of faculty’s abilities and skills, payment pattern according to the teachers’ quality and quantity performance and community situations, fellowships and creating opportunities for faculty participation in decision making. Also, it is recommended that some interventions should be planned concerning the improving quality of work life and its efficiency should be evaluated.
